# Effect of Ivermectin and Atorvastatin on Nuclear Localization of Importin Alpha and Drug Target Expression Profiling in Host Cells from Nasopharyngeal Swabs of SARS-CoV-2- Positive Patients

**DOI:** 10.3390/v13102084

**Published:** 2021-10-15

**Authors:** Valeria Inés Segatori, Juan Garona, Lorena Grisel Caligiuri, Juan Bizzotto, Rosario Lavignolle, Ayelén Toro, Pablo Sanchis, Eduardo Spitzer, Alejandro Krolewiecki, Geraldine Gueron, Daniel Fernando Alonso

**Affiliations:** 1 Centro de Oncología Molecular y Traslacional y Plataforma de Servicios Biotecnológicos, Departamento de Ciencia y Tecnología, Universidad Nacional de Quilmes, Bernal B1876BXD, Argentina; valeria.segatori@unq.edu.ar (V.I.S.); juan.garona@unq.edu.ar (J.G.); lorena.caligiuri@unq.edu.ar (L.G.C.); 2 Centro de Medicina Traslacional, Hospital El Cruce, Florencio Varela B1888AAE, Argentina; 3 Departamento de Química Biológica, Facultad de Ciencias Exactas y Naturales, Universidad de Buenos Aires, Biológica, Intendente Guiraldes 2160, Buenos Aires C1428EGA, Argentina; juanantoniobizzotto@gmail.com (J.B.); rosario.lavignolle@gmail.com (R.L.); ayelentoro@gmail.com (A.T.); pabloasanchis@gmail.com (P.S.); 4 CONICET—Universidad de Buenos Aires, Instituto de Química Biológica de la Facultad de Ciencias Exactas y Naturales, Buenos Aires C1428EGA, Argentina; 5 Laboratorio Elea-Phoenix, Los Polvorines B1613AUE, Argentina; eduardo.spitzer@elea.com; 6 Instituto de Investigaciones de Enfermedades Tropicales (IIET-CONICET), Sede Regional Orán, Universidad Nacional de Salta, Orán A4530ANQ, Argentina; alekrol@hotmail.com

**Keywords:** SARS-CoV-2, COVID-19, drug repurposing, antihelmintic drug, lipophilic statin, host cell antiviral response, ivermectin, atorvastatin

## Abstract

Nuclear transport and vesicle trafficking are key cellular functions involved in the pathogenesis of RNA viruses. Among other pleiotropic effects on virus-infected host cells, ivermectin (IVM) inhibits nuclear transport mechanisms mediated by importins and atorvastatin (ATV) affects actin cytoskeleton-dependent trafficking controlled by Rho GTPases signaling. In this work, we first analyzed the response to infection in nasopharyngeal swabs from SARS-CoV-2-positive and -negative patients by assessing the gene expression of the respective host cell drug targets importins and Rho GTPases. COVID-19 patients showed alterations in KPNA3, KPNA5, KPNA7, KPNB1, RHOA, and CDC42 expression compared with non-COVID-19 patients. An in vitro model of infection with Poly(I:C), a synthetic analog of viral double-stranded RNA, triggered NF-κB activation, an effect that was halted by IVM and ATV treatment. Importin and Rho GTPases gene expression was also impaired by these drugs. Furthermore, through confocal microscopy, we analyzed the effects of IVM and ATV on nuclear to cytoplasmic importin α distribution, alone or in combination. Results showed a significant inhibition of importin α nuclear accumulation under IVM and ATV treatments. These findings confirm transcriptional alterations in importins and Rho GTPases upon SARS-CoV-2 infection and point to IVM and ATV as valid drugs to impair nuclear localization of importin α when used at clinically-relevant concentrations.

## 1. Introduction

Since the early days of the coronavirus disease 2019 (COVID-19) pandemic, drug repurposing has aroused great interest in the development of novel antiviral therapies. This concept, also called rediscovering or therapeutic indication expansion, implies establishing new medical uses for already known drugs [1]. Different agents have been investigated for antiviral activity against severe acute respiratory syndrome coronavirus 2 (SARS-CoV-2) including antimalarials, antiparasitics, antibiotics, antihypertensives, and hypoglycemics [2].

Many drugs with potential effects in COVID-19 infection have also been previously explored as repurposed options for cancer treatment, and suggestive similarities were found to exist between antitumor and host-based antiviral mechanisms [3]. Clinically approved drugs could be used to target intracellular mechanisms in host cells that are essential for viral replication. In this regard, nuclear transport and vesicle trafficking are key cellular functions involved in the pathogenesis of RNA viruses and also during cancer development.

Ivermectin (IVM) is a well-known antihelmintic drug with reported antiviral activity against Dengue, Zika and Influenza virus [4]. Among the pleiotropic effects on virus-infected host cells, IVM is capable of interfering with importin α/β-mediated nuclear transport, thus inhibiting nuclear import mechanisms of viral proteins and reducing viral replication [5]. Early in the COVID-19 pandemic, Caly et al. communicated a potent activity of high concentrations of IVM (IC50 of about 2.5 µM) against SARS-CoV-2 in Vero cell cultures [6]. However, a question remains whether these high concentrations can be effectively achieved clinically at safe doses. Recently, we reported the results of a proof-of-concept randomized clinical trial in hospitalized COVID-19 patients receiving high oral doses of IVM (0.6 mg/kg/day) [7]. Although no differences between treated and control patients were observed in SARS-CoV-2 viral load, a concentration-dependent antiviral activity was noticed after five days of IVM treatment. A relationship was identified between high IVM plasma concentrations (>160 ng/mL) and significant increases of SARS-CoV-2 viral decay rates in respiratory secretions [7].

Beyond the obvious importance of dosing schedules and tissue concentrations of IVM, it is also attractive to search for drug combinations that could potentiate its antiviral mechanism in host cells. Lipophilic statins used in the treatment of hypercholesterolemia such as atorvastatin (ATV) are well-tolerated compounds with antitumor activity that are interesting host-based drug candidates for SARS-CoV-2 in combination with IVM [3]. It is known that statins reduce membrane localization of Rho GTPases, thus affecting intracellular signaling involved in the organization of actin cytoskeleton during the metastatic process [8]. Interestingly, the Rho GTPases Rho A and CDC42 are also responsible for actin-dependent host cell protein trafficking, playing important roles in the intracellular transport of viral proteins [9].

In the present work, we first analyzed susceptibility infection profiles in SARS-CoV-2-positive versus -negative patients in a case-control study (GSE152075), evaluating the expression of the respective host cell drug targets importins and Rho GTPases. We then modeled the infection in vitro using Poly(I:C), a synthetic analog of viral double-stranded RNA, assessing IVM and ATV effects on drug targets at the transcriptional level. Finally, we conducted a confocal microscopy study to analyze the in vitro effects of IVM on nuclear localization of importin α, alone and in combination with ATV, at potentially clinically-relevant concentrations.

## 2. Materials and Methods

### 2.1. Analysis of Publicly Available RNA-Seq Datasets

RNA-Seq data analysis from COVID-19 and non-COVID-19 patients was performed as previously described [10]. We downloaded the GSE152075 dataset [11], which contains pseudoaligned pre-processed RNA-Seq data and clinico-pathological information from 430 SARS-CoV-2 positive and 54 negative patients.

For RNA-Seq analysis from in vitro and in vivo experiments, we used GSE147507 dataset [12], which contains RNA-Seq data from: (i) independent biological triplicates of transformed lung alveolar cells (A549) transduced with a vector expressing human ACE2 infected with SARS-CoV-2 (MOI: 2) or mock-phosphate-buffered saline (PBS); and (ii) tracheal samples from 4-month-old ferrets intranasally infected with 5 × 10^4^ PFU of SARS-CoV-2 or mock-PBS.

We removed samples with >70% of total genes with 0 sequence reads, considering them as very low-quality samples that might introduce a bias. Normalization, batch effect correction, and differential expression were performed with R package DEseq2 v1.28.1 [13]. Age was categorized according to the WHO guidelines [14]: <30 years old, every 10 years between 30 and 70 years old, and ≥70 years old as described [15]. To stratify COVID-19 positive patients based on viral load at the time of diagnosis, we used the PCR cycle threshold (Ct) of the N1 viral gene amplification as a surrogate variable for viral load (Ct > 24 = low; Ct = 24–19 = medium; Ct < 19 = high).

Microarray analysis from human microvascular endothelial cells (HMVEC) treated or not with ATV (10 µM 24 h) were performed using differential expression data from the GSE8686 dataset [16].

### 2.2. Drugs and Reagents

IVM (MW 875 g/mol; PubChem ID 24278497) and ATV calcium trihydrate (MW 1209.4 g/mol; PubChem ID 656846) were obtained from Elea-Phoenix (Buenos Aires, Argentina), following the Good Manufacturing Practices standards (lot numbers 227974 and 223557, respectively). Compounds were first resuspended in dimethyl sulfoxide (DMSO) generating concentrated stocks of 11.4 and 10 mM, respectively, and aliquoted at −20 °C. Final working concentrations were achieved after serial dilutions using PBS. Control groups were treated with PBS plus corresponding DMSO concentrations as the vehicle. Immunolabeling of cells was conducted using a primary anti-importin α (KPNA2) antibody generated in rabbit at 1/250 dilution and a secondary polyclonal anti-rabbit IgG generated in goat conjugated with Alexa 594 at 1/200 dilution (Abcam, Cambridge, UK). High molecular weight Poly(I:C) was purchased from InvivoGen (San Diego, CA, USA) and prepared according to manufacturer’s instructions. Briefly, endotoxin-free water was added to Poly(I:C) at a final concentration of 1 mg/mL, incubated at 65 °C for 10 min, aliquoted, and stored at −20 °C until use.

### 2.3. Cell Lines and Culture Conditions

Human cervix adenocarcinoma HeLa (ATCC^®^ CCL-2™), human lung carcinoma A549 (ATCC^®^ CCL-185™), and green monkey kidney epithelium Vero (ATCC^®^ CCL-81™) cells were grown in Dulbecco’s modified Eagle’s medium (DMEM, Gibco, Rockville, MD, USA) plus 10% fetal bovine serum (FBS), 2 mM glutamine, and 80 μg/mL gentamycin in a monolayer culture, at 37 °C in a humidified atmosphere of 5% CO_2_. Cells were harvested using a trypsin/EDTA solution (Gibco) diluted in PBS and routinely tested for mycoplasma.

### 2.4. Poly(I:C) Treatment

Poly(I:C) intracellular administration was performed by transfection with Lipofectamine LTX (Invitrogen, Carlsbad, CA, USA) according to the manufacturer’s instructions at a final concentration of Poly(I:C) of 10 µg/mL. Briefly, A549 cells were plated in 6-well flat bottom plates at a density of 2 × 10^5^ cells in complete DMEM, allowed to attach overnight, and then transfected with Poly(I:C) or water as mock in DMEM without FBS and antibiotics. After 6 h, the media were replaced and cells were treated with IVM (2.5 µM) and ATV (10 µM) alone or in combination.

### 2.5. RNA Isolation, c-DNA Synthesis, and Quantitative Real-Time PCR (RT-qPCR)

Total RNA was isolated with Quick-Zol (Kalium technologies, Buenos Aires, Argentina) according to the manufacturer’s protocol. cDNAs were synthesized with RevertAid Premium First Strand cDNA Synthesis Kit (Fermentas, Waltham, MA, USA) and used for real-time PCR amplification with Taq DNA Polymerase (Invitrogen, Waltham, MA, USA) in a QuantStudio 3 Real-Time PCR System (Thermo Fisher Scientific, Waltham, MA, USA). *PPIA* was used as the internal reference gene. Data were analyzed using the method of 2-∆∆CT [17]. Table 1 describes the forward and reverse primer sequences designed specifically for each species to avoid cross-reactivity.

### 2.6. Cell Viability Assay

Cytostatic effects of IVM on Hela and Vero cells were measured using the 3-(4,5-dimethylthiazol-2-yl)-2,5 diphenyltetrazolium bromide (MTT) assay (Sigma-Aldrich, St Louis, MO, USA) [18]. Briefly, cells were plated in 96-well flat bottom plates at a density of 5 × 10^3^ cells per 200 µL in complete D-MEM, allowed to attach overnight, and then treated for 48 h with increasing concentrations of IVM (2.5, 5, and 25 µM). After adding 20 µL of MTT reagent to each well, plates were incubated for 2.5 h. Formazan crystals were solubilized using DMSO and the absorbance of each well was measured at 570 nm. The optical density of the vehicle-treated control cells was taken as 100% viability.

### 2.7. Immunofluorescence Staining of Importin α

Briefly, 2 × 10^4^ cells were seeded on glass coverslips in 24-well flat bottom plates and cultured for 48 h in complete growth medium. After reaching ≈40% confluence, cells were exposed to different drug concentrations for 1–24 h and then fixed with 10% neutral buffered formalin for 10 min at 4 °C. After permeabilizing and blocking with 0.2% Triton X-100 and 4% FBS in PBS, respectively, cells were incubated with a primary rabbit anti-Importin α antibody for 1 h at 37 °C. Importin α-bound antibodies were detected with a secondary goat polyclonal Alexa 594-conjugated antibody for 40 min at room temperature and nuclei were labeled with DAPI (Vector Laboratories, Peterborough, UK) [19]. Absence of immunofluorescence staining was confirmed when the primary antibody was omitted.

### 2.8. Confocal Microscopy Studies and Acquisition Settings

Nuclear/cytoplasmic importin α distribution in Hela and Vero cells was assessed using a TCS SP8 confocal laser scanning microscope (CLSM, Leica Microsystems, Wetzlar, Germany). CLSM was equipped with a 405 nm diode and 561 nm helium-neon lasers and operated by the Leica Application Suite X program (LAS X V3.7.2, Leica Microsystems). Twelve-bit images with 1024 × 1024-pixel resolution were obtained by sequential scanning using a 63× oil immersion objective. Pinhole and gain values were set at 1 and 600, respectively. LAS X software was used for the analysis of digitized CLSM images in order to calculate the nuclear/cytoplasmic fluorescence ratio (Fn/Fc), following the formula: Fn/Fc = (Fn − Fb)/(Fc − Fb), where Fb is the background autofluorescence [5,20,21]. Two independent trained operators used a semi-automated hand-drawn polygon contouring system to delineate specific ROIs for nuclear or cytoplasmic fluorescence quantification [22]. Single cell values were averaged for a minimum of 40 cells per experimental condition as a result of two or three independent experiments to correct for variations in expression levels [23,24].

### 2.9. Statistics

To determine statistical differences between categorical groups in gene expression analysis, we performed two-tailed Welch’s *t* tests when the assumption normality was maintained and Wilcoxon rank sum test when we could not assume normal distribution. Two-sided, increasing and decreasing Jonckheere–Terpstra trend tests (with 500 permutations) were used to determine statistical trends between gene expression and age groups. To study pairwise correlations between continuous variables, the Spearman’s rank correlation coefficient was calculated. We did not correct the *p*-value for multiple testing. All results were plotted using ggplot2 [25], ggpubr [26], and GGally [27] in R [28].

For confocal microscopy and cellular studies, in order to compare differences between two experimental groups, two-tailed Mann–Whitney or t-tests were used for non-parametric or normal distribution of data, respectively. In case of more than two experimental groups, ANOVA analysis with Tukey’s multiple comparisons post-test was used. Kruskal–Wallis analysis with Dunn’s multiple comparisons post-test was used in the case of non-parametric distribution of data. Data were derived from at least two or three independent experiments, unless stated otherwise. Data were presented as mean ± standard error of mean (SEM), scattered dot blot with median ± quartiles, or violin plots.

Differences were considered statistically significant at a level of *p* < 0.05. Data processing and statistical analysis was performed using the Prism 6.1 Software (GraphPad Holdings, CA, USA).

## 3. Results

### 3.1. Expression of the Importin Family Genes in SARS-CoV-2-Positive and -Negative Patients

**Figure 1:**
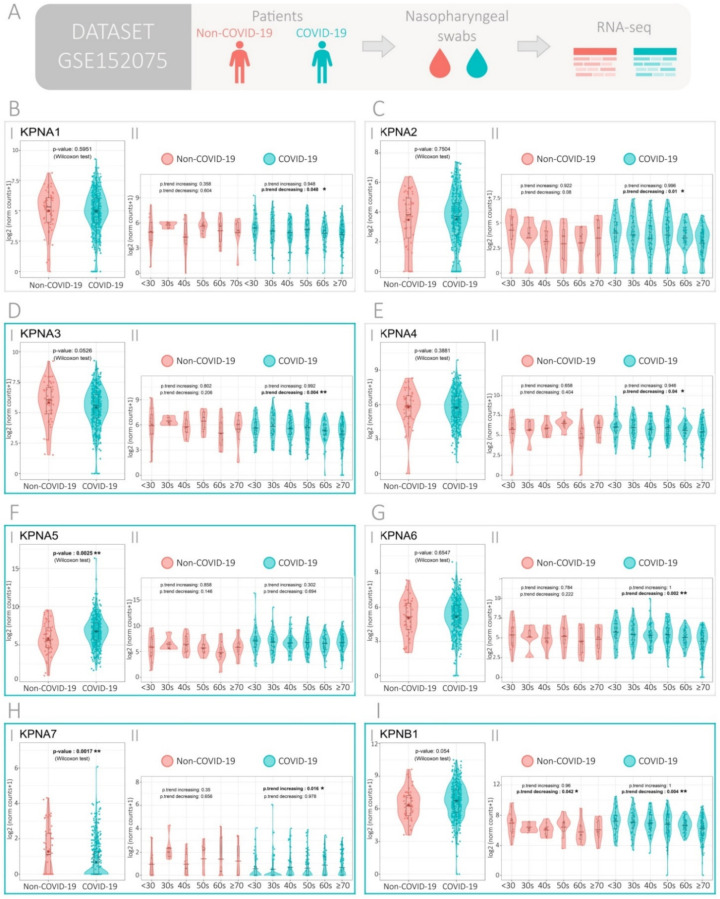
Expression of the importin family genes in COVID-19 and non-COVID-19 patients. (**A**) Experimental design of the GSE152075 dataset, composed of transcriptome data from nasopharyngeal swabs collected from 430 COVID-19 and 54 non-COVID-19 patients. (**B**–**I**) Gene expression analysis for importin genes (**B**) *KPNA1*, (**C**) *KPNA2*, (**D**) *KPNA3*, (**E**) *KPNA4*, (**F**) *KPNA5*, (**G**) *KPNA6*, (**H**) *KPNA7*, and (**I**) *KPNB1*. Each gene is divided in two panels: (I) COVID-19 versus non-COVID-19 patients (*p*-values correspond to Wilcoxon rank-sum test, except when the assumption normality was maintained, then two-tail Welch’s *t* test was used). (II) COVID-19 and non-COVID-19 patients categorized by age groups (*p*-values correspond to increasing and decreasing Jonckheere–Terpstra trend tests). Blue boxes indicate genes with significant or marginal significant *p*-value when comparing gene expression between COVID-19 and non-COVID-19 patients.

We used the publicly available RNA sequencing dataset GSE152075 to evaluate the expression of the importin family genes (Importin α5 (*KPNA1*), Importin α1 (*KPNA2*), Importin α4 (*KPNA3*), Importin α3 (*KPNA4*), Importin α6 (*KPNA5*), Importin α7 (*KPNA6*), Importin α8 (*KPNA7*), and Importin β1 (*KPNB1*)) in COVID-19 positive and negative patients (Figure 1A). This set contained transcriptomic data from nasopharyngeal swabs from 430 SARS-CoV-2-positive and 54 SARS-CoV-2-negative patients. Patient demographics are available in Table S1.

Results showed that COVID-19 patients had higher KPNA5 (Figure 1F.I) and lower KPNA7 (Figure 1H.I) expression compared with non-COVID-19 patients (*p* = 0.0025 and, *p* = 0.0017, respectively). Decrease in KPNA3 (*p* = 0.0526) and increase in KPNB1 (*p* = 0.054) expressions were found with marginal significance in COVID-19 patients (Figure 1D.I,I.I). No statistical differences for the other importin genes were observed between COVID-19 and non-COVID-19 patients (Figure 1A.I,B.I,C.I,E.I,G.I).

Since age and sex have been reported as risk factors for SARS-CoV-2 infection, we additionally assessed the association between the importins’ gene expression and these risk factors. When assessing gene expression in patients categorized by age, we found that age increase was significantly associated with lower KPNA2, KPNA3, KPNA4, KPNA6, and KPNB1 expression (*p*-trend decreasing = 0.01; *p*-trend decreasing = 0.004; *p*-trend decreasing = 0.04, *p*-trend decreasing = 0.002; *p*-trend decreasing = 0.004 respectively) and higher KPNA7 expression in COVID-19 patients (*p*-trend increasing = 0.016), but not in non-COVID-19 patients (Figure 1B(I,II)). No statistical differences were found when comparing between male and female patients (Figure S1A). To further our analysis, we categorized patients based on their viral load at time of diagnosis. We used the PCR cycle threshold (Ct) of the N1 viral gene amplification as a surrogate variable for viral load (Figure S2). The analysis showed similar results to the unstratified analysis.

Results were then validated in the GSE147507 dataset, which covers transcriptomic data from A549 cells infected or not with SARS-CoV-2 as well as data from trachea biopsies of 4-month-old ferrets harvested three days after SARS-CoV-2 infection or mock treatment (Figure S3A). When analyzing gene expression in A549 cells, results showed a decrease in *KPNA2* (*p* = 0.0285) and an increase in *KPNA5* (*p* = 0.0266) upon viral infection (Figure S3B). Additionally, ferret infection with SARS-CoV-2 showed an increase in *KPNA1*, *KPNA4*, and *KPNA5* expression (*p* = 0.0473, *p* = 0.0449, and *p* = 0.0371, respectively) when compared with the mock treatment (Figure S3C). Interestingly, *KPNA5* appears upregulated upon infection, across the different datasets.

### 3.2. Expression of the Rho GTPase Family Genes in SARS-CoV-2-Positive and -Negative Patients

Since viral infection relies on intra-cellular protein transport modulated by Rho GTPases, we sought to evaluate changes in the gene expression of these proteins upon SARS-CoV-2 infection. The Rho GTPase genes Ras Homolog Family Member A (*RHOA*), Rac Family Small GTPase 1 (*RAC1*) and Cell Division Cycle 42 (*CDC42*) were assessed.

**Figure 2:**
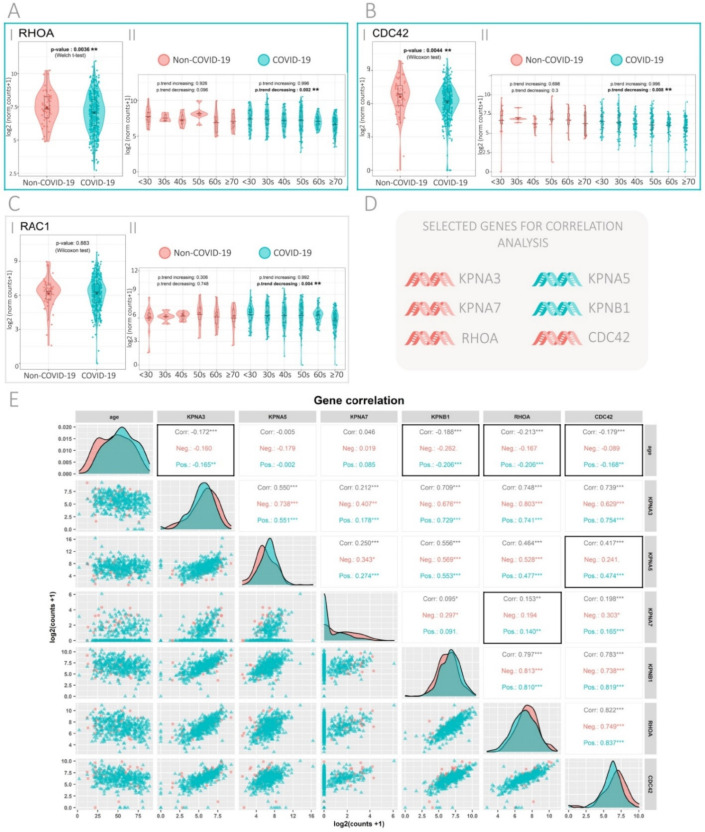
Expression of the Rho GTPase family genes in COVID-19 and non-COVID-19, and correlation analysis. (**A**–**C**) Gene expression analysis for Rho GTPase genes (**A**) *RHOA*, (**B**) *CDC42*, and (**C**) *RAC1*. Each gene is divided in two panels: (I) COVID-19 versus non-COVID-19 patients (*p*-values correspond to Wilcoxon rank-sum test, except when the assumption normality was maintained, then two-tail Welch’s t test was used). (II) COVID-19 and non-COVID-19 patients categorized by age groups (*p*-values correspond to increasing and decreasing Jonckheere–Terpstra trend tests). Light green boxes indicate genes with significant or marginal significant *p*-value when comparing gene expression between COVID-19 and non-COVID-19 patients. (**D**) Summary of importin and Rho GTPase genes selected for correlation analysis. Red color represents genes that are downregulated and light green represents genes that are upregulated in COVID-19 and non-COVID-19 patients. (**E**) Pairwise Spearman correlation matrix analysis between age, *KPNA3*, *KNA5, KPNA7*, *KPNB1*, *RHOA*, and *CDC42*. The upper half displays the Spearman coefficients (r) considering all patients (Corr.), non-COVID-19 patients (Neg.), or COVID-19 patients (Pos.). Black boxes highlight genes that have significant correlation in COVID-19, but not in non-COVID-19 patients. * *p* < 0.05; ** *p* < 0.01; *** *p* < 0.001. The lower half displays the scatterplots.

Results show that *RHOA* and *CDC42* expression was significantly decreased (*p* = 0.0036, *p* = 0.0044, respectively) in COVID-19 compared with non-COVID-19 patients (Figure 2A.I,B.I). All three Rho GTPases, *RHOA*, *CDC42*, and *RAC1*, showed a significant decrease in gene expression upon age in COVID-19 patients (Figure 2A.II,B.II,C.II; *p*-trend decreasing = 0.002, *p*-trend decreasing = 0.008, *p*-trend decreasing = 0.004, respectively). No statistical differences were found when comparing between male and female patients (Figure S1B).

Regarding the infection in A549 cells, no changes were observed when assessing gene expression of the Rho GTPase family compared with the controls (Figure S3B). The ferret infection dataset did not provide RNA-Seq reads for the Rho GTPases.

### 3.3. Gene Correlation Analysis in SARS-CoV-2-Positive and -Negative Patients

Next, pairwise Spearman correlation analyses were performed between genes that had significant or marginal significant changes in gene expression upon infection with SARS-CoV-2 in patients (Figure 2D). Patient age was also correlated with gene expression. For each combination of variables, analyses were performed for all patients, COVID-19 patients only and non-COVID-19 patients only (Figure 2E). Black boxes indicate combinations of variables where correlation was significant for COVID-19 patients, but not for non-COVID-19. Interestingly, *RHOA* with *KPNA7* (r = 0.140, *p* < 0.01) and *CDC42* with *KPNA5* (r = 0.474, *p* < 0.001) showed positive correlations compared with no significant correlations in non-COVID-19 patients.

### 3.4. Modulation of Importin and Rho GTPases Transcriptional Expression by IVM and ATV in a Viral Infection Simulation Context

To further our analysis, we used Poly(I:C), a synthetic dsRNA compound that binds Toll- like receptor 3 (TLR3), to mimic a viral infection. Poly(I:C) recapitulates many of the effects observed in viral dsRNA in vitro and in vivo [29]. In this model, we examined the effect of IVM and ATV on importin family and Rho GTPases gene expression in A549 cells. We selected the A549 cell line as the lung is the principal homing organ for SARS-CoV-2 [30]. Cells were treated with or without Poly(I:C) and then subjected to high IVM concentration (2.5 µM for 1 h) alone or in combination with ATV (10 µM for 24 h).

**Figure 3:**
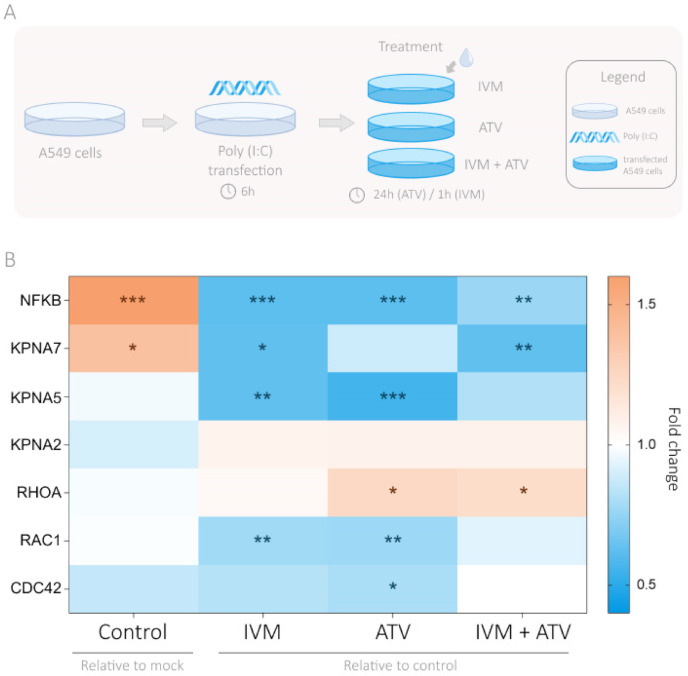
Dysregulation of importin and Rho GTPases gene expression in A549 cells transfected with Poly(I:C) and treated with IVM and ATV. (**A**) Experimental design of IVM and ATV treatment in a viral infection simulation context. (**B**) Heatmap depicting *NFKB, KPNA7*, *KPNA5*, *KPNA2*, *RHOA*, *RAC1*, and *CDC42* fold changes in mRNA levels assessed by real time PCR (RT-qPCR) in A549 cells transfected with Poly(I:C) (10 µg/mL) and treated with PBS as the control, IVM (2.5 µM), ATV (10 µM), or the combination of both drugs (IVM + ATV). Values were normalized using PPIA as a reference gene. Gene expression for control Poly(I:C) transfected cells was relativized to mock condition. Gene expression for IVM, ATV, and IVM + ATV treated cells was relativized to the control Poly(I:C) condition. Red, white, and blue represent a fold change >1, fold change = 1 or fold change <1, respectively. * *p* < 0.05; ** *p* < 0.01; *** *p* < 0.001.

As shown in Figure 3, Poly(I:C) treatment increased NFKB expression, validating the Poly(I:C) responsiveness of A549 cells. Regarding importin genes, *KPNA7* and *KPNA5* expressions were reduced in the presence of IVM and ATV alone or in combination. Particularly, *KPNA7* expression was significantly decreased by IVM and IVM combined with ATV while *KPNA5* expression was significantly lower with ATV and IVM alone. *KPNA2* expression was not altered by either Poly(I:C) stimulation or ATV and IVM treatments (Figure 3 and Figure S3A). Regarding Rho GTPases, gene expression of *RHOA*, *RAC1*, and *CDC42* was not altered by Poly(I:C). ATV treatment, alone or in combination, increased *RHOA* expression (*p* < 0.05 and *p* < 0.05, respectively). Conversely, *RAC1* expression was significantly downregulated in the presence of ATV (*p* < 0.01) or IVM (*p* < 0.01) while *CDC42* expression was significantly decreased by ATV (*p* < 0.05) (Figure 3 and Figure S4A). In parallel, we assessed the GSE8686 dataset that comprises human microvascular endothelial cells (HMVEC) treated or not with the same concentration of ATV (10 µM 24 h). The data reflect a significant downregulation of *RHOA* and *RAC1* at the transcriptional level (Figure S4B).

### 3.5. Effect of IVM and ATV on Importin α Nuclear Accumulation

**Figure 4:**
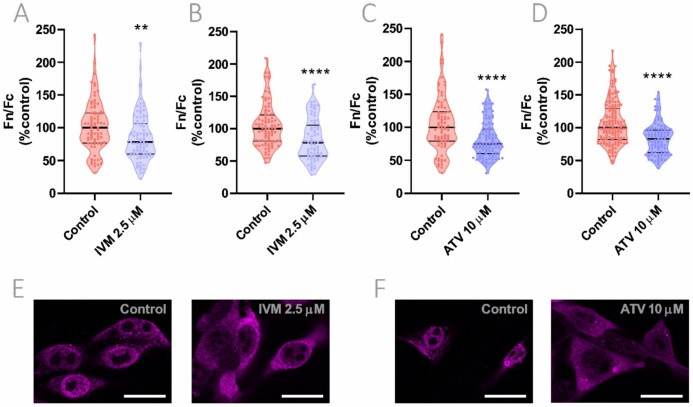
Effect of IVM and ATV on importin α nuclear accumulation. (**A**–**D**). Importin α nuclear to cytoplasmic distribution was analyzed by confocal microscopy in Hela (**A**,**C**) and Vero (**B**,**D**) cells treated with 2.5 μM IVM for 1 h (**A**,**B**) or 10 μM ATV for 24 h (**C**,**D**). Each data point represents Fn/Fc from a single cell; data were normalized to control cells and median ± quartiles are indicated. ** *p* < 0.01; **** *p* < 0.0001; Mann–Whitney test. (**E**,**F**) Representative confocal images of Hela cells exposed to IVM (**E**) or ATV (**F**) showing the effect of the drugs on importin α cellular distribution after treatment. Scale bar = 20 μm.

Next, we sought to evaluate the effect of IVM on importin α cellular distribution. Cells were exposed to 2.5 μM IVM for 1 h and nuclear to cytoplasmic importin α distribution was evaluated in Hela and Vero cells by confocal microscopy. IVM significantly reduced nuclear accumulation in both cell lines, showing a 20% decrease in comparison to vehicle-treated cells (*p* < 0.05; *p* < 0.0001, respectively) (Figure 4A,B). Representative confocal images of Hela cells are shown in Figure 4C. To discard any direct cytotoxic effects of the compound, we evaluated cell viability after 48 h-incubation with increasing concentrations of IVM. As shown in Figure S5, there were no signs of cytotoxicity when cells were treated with 2.5 µM IVM. Additionally, as a positive control of importin α nuclear accumulation, cells were exposed to an oxidative stress condition induced by hydrogen peroxide [31]. After 1 h-incubation using 200 μM of hydrogen peroxide, a significant increase in 30% in importin α nuclear accumulation was observed (*p* < 0.05) (Figure S6).

As previously mentioned, ATV is a lipophilic statin that affects actin cytoskeleton organization and, as a consequence, is known to alter protein transport in host cells after 24 h-treatment [8,32]. In this context, we evaluated if ATV was particularly capable of altering importin α cellular distribution in Hela and Vero cells. Long-term treatment with 10 μM ATV during 24 h caused a significant reduction in importin α nuclear accumulation in both cell lines, decreasing its nuclear to cytoplasmic localization ratio by 25 and 17%, respectively (*p* < 0.0001) (Figure 4D,E). Representative confocal images of ATV-treated Hela cells are shown in Figure 4F. Short-term exposure to ATV during 1 or 6 h did not have any impact on importin α cellular distribution (Figure S7).

### 3.6. Combinational Effect of IVM Plus ATV Treatment on Importin α Nuclear Accumulation

**Figure 5:**
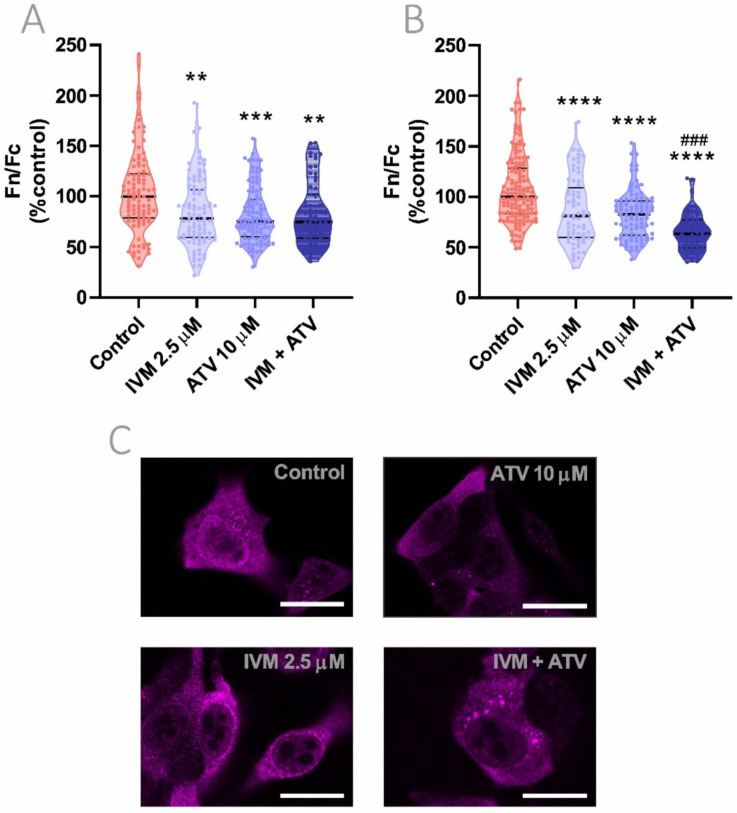
Evaluation of importin α nuclear accumulation after combination treatment using IVM plus ATV. (**A**) Hela and (**B**) Vero cells were treated with 2.5 μM IVM, 10 μM ATV, or IVM + ATV and importin α cellular distribution was evaluated by confocal microscopy. Each data point represents Fn/Fc from a single cell; data were normalized to control cells and median ± quartiles are indicated. ** *p* < 0.01; *** *p* < 0.001; **** *p* < 0.0001 vs. control group; ### *p* < 0.001 vs. IVM and ATV monotherapies; Kruskal–Wallis followed by Dunn’s multiple comparisons test. (**C**) Representative confocal images of Hela cells treated with IVM, ATV, and IVM + ATV showing a combination treatment effect on importin α nuclear localization. Scale bar = 20 μm.

Considering the effect on protein trafficking in general and on importin α cellular distribution in particular, the combination of IVM and ATV appears to be an interesting approach in order to achieve greater host-mediated antiviral responses. We further evaluated the effect of ATV addition to previously tested IVM concentration (2.5 µM for 1 h). In both cell lines, the combined treatment resulted in a significant decrease in nuclear to cytoplasmic importin α distribution when compared to vehicle-treated cells (*p* < 0.01, Hela; *p* < 0.0001, Vero). Even though in Hela cells the combination of IVM and ATV had an equivalent effect on importin α nuclear accumulation than individual treatments, in Vero cells, combined treatments showed an additive inhibition of importin α nuclear accumulation when compared to each monotherapy (*p* < 0.001) (Figure 5A,B). Additionally, it is worth noting that the reduction in nuclear to cytoplasmic importin α distribution in treated cells was accompanied by a consistent increase in perinuclear importin α accumulation. This perinuclear rim pattern was observed especially in IVM-treated cells, alone or plus ATV, being possibly related to impaired importin heterodimer formation in the nuclear proximity.

### 3.7. Reduction of Importin α Nuclear Accumulation Using a >10-Fold Lower IVM Concentration

**Figure 6:**
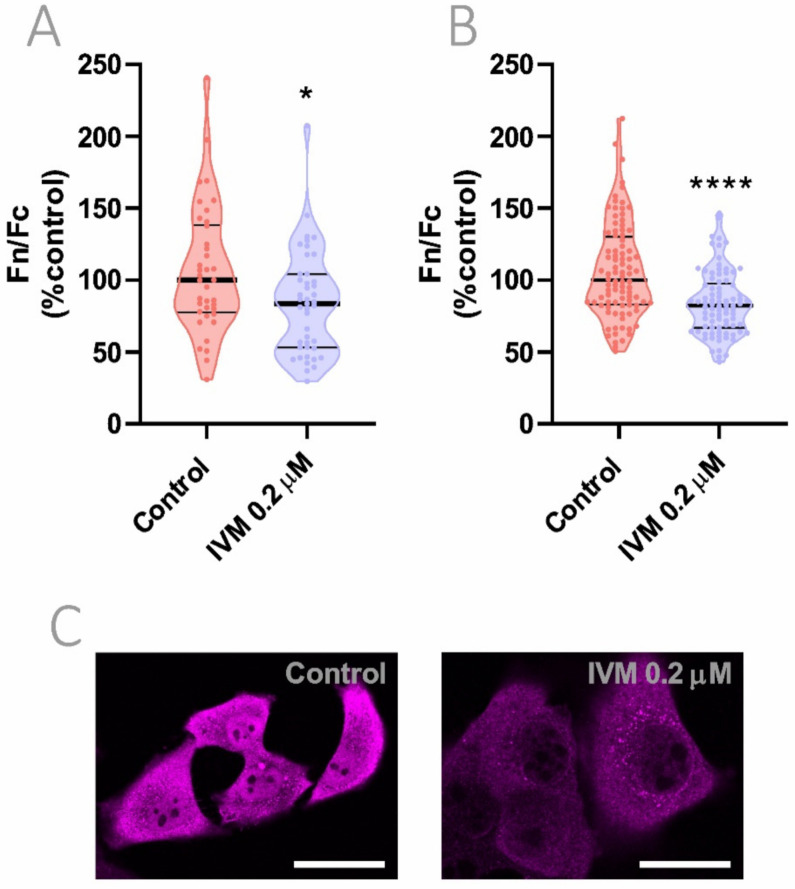
Effect of IVM at low concentration on importin α nuclear accumulation. (**A**,**B**) Importin α nuclear to cytoplasmic distribution evaluated in Hela (**A**) and Vero (**B**) cells treated with 0.2 μM IVM for 24 h. Each data point represents Fn/Fc from a single cell; data were normalized to control cells and median ± quartiles are indicated. * *p* < 0.05; **** *p* < 0.0001; Mann–Whitney test. (**C**) Representative confocal images of Hela cells treated with 0.2 μM IVM. Scale bar = 20 μm.

Recent results of our proof-of-concept randomized clinical trial showed a relationship between drug exposure and antiviral activity in patients with plasma IVM concentrations of 160 ng/mL or higher [7]. Taking into account that the equivalent concentration for in vitro cell treatment results in approximately 0.2 μM, we analyzed the biological effect of this achievable, safe, and clinically-relevant concentration. As shown in Figure 6, exposure for 24 h to an IVM concentration of 0.2 μM caused a similar decrease in importin α nuclear accumulation compared to a high IVM concentration of 2.5 μM for 1 h, reducing nuclear to cytoplasmic importin α distribution by nearly 20% in both Hela (Figure 6A) and Vero (Figure 6B) cell lines (*p* < 0.05; *p* < 0.0001, respectively).

As importin α is involved in the nuclear transport of different cargo proteins, we additionally evaluated the functional impact of low concentration IVM on the cellular distribution of one of its reported cargo proteins [5]. As observed in Figure S8, sustained exposure to 0.2 μM IVM was also associated with a significant reduction in phosphorylated-p53 nuclear import (*p* < 0.05).

### 3.8. Reduction of Importin α Nuclear Accumulation Using IVM at Low Concentration in Combination with ATV

**Figure 7:**
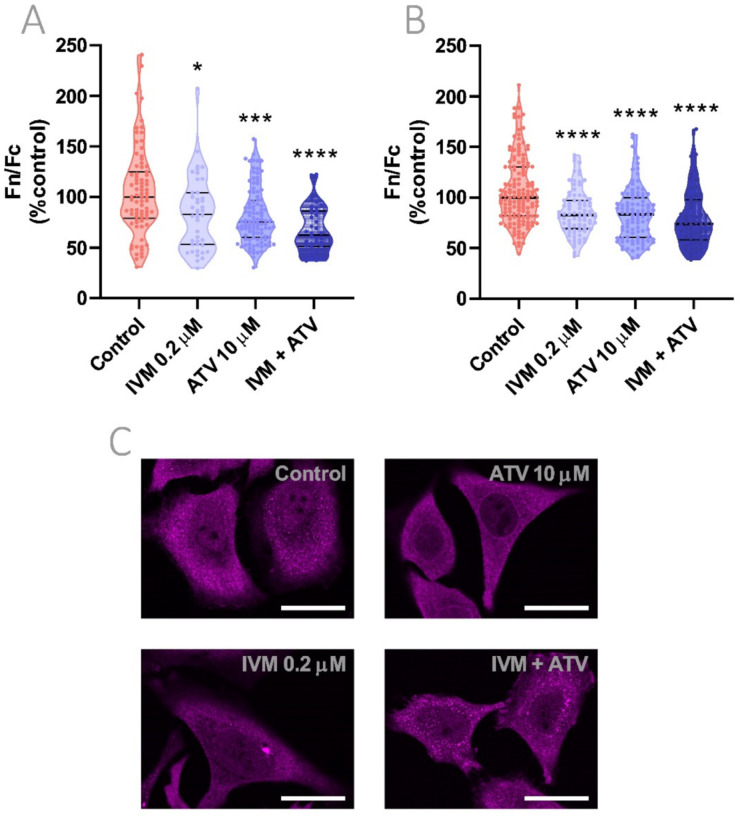
Evaluation of importin α nuclear accumulation after combination treatment using IVM at low concentration plus ATV. (**A**) Hela and (**B**) Vero cells were treated with 0.2 μM IVM, 10 μM ATV, or IVM + ATV and importin α cellular distribution was evaluated by confocal microscopy. Each data point represents Fn/Fc from a single cell; data were normalized to control cells and median ± quartiles are indicated. * *p* < 0.05; *** *p* < 0.001, **** *p* < 0.0001 vs. control group; Kruskal–Wallis followed by Dunn’s multiple comparisons test. (**C**) Representative confocal images of Hela cells treated with IVM, ATV, or IVM + ATV showing a combination treatment effect on importin α nuclear localization when low concentration IVM was used. Scale bar = 20 μm.

We finally evaluated the addition of ATV to the low IVM concentration of 0.2 μM during 24 h. As observed in Figure 7, in all evaluated experimental settings nuclear to cytoplasmic importin α distribution was significantly reduced when compared to vehicle-treated cells (*p* < 0.0001). Despite not reaching statistical significance against IVM or ATV monotherapies, larger reductions in importin α nuclear accumulation were observed after IVM plus ATV combined treatments, showing a 38 or 25% reduction in nuclear to cytoplasmic importin α localization ratio in the Hela (Figure 7A) or Vero (Figure 7B) cells, respectively.

## 4. Discussion

In this work, we have ascertained in patient samples, in an animal model, and in cell cultures in vitro, significant alterations of the importin family at the transcriptional level upon SARS-CoV-2 infection. Furthermore, IVM and ATV had significant effects on the transcriptional activity of importins and Rho GTPases upon mimicked viral infection. However, the main proposed antiviral mechanism of IVM in host cells is thought to be its ability to interfere with the nuclear transport of viral proteins mediated by importins [4,5,33].

A potent antiviral activity in vitro against SARS-CoV-2 has been communicated using high IVM concentrations of 2.5 µM [6]. Such concentrations correspond to a plasma concentration of about 2200 ng/mL, which is much higher than the peak plasma level normally expected with the standard ivermectin oral dose of 0.2 mg/kg [34,35]. Thus, the initial enthusiasm for the potential use of IVM in COVID-19 treatment turned into skepticism based on pharmacology data, suggesting that effective tissue concentrations may not be achievable in humans. However, IVM is characterized by a wide therapeutic index, with doses up to 10 times those usually prescribed for antihelmintic indications (2 mg/kg), demonstrating good safety and tolerability in healthy volunteers [36].

In the present study, we demonstrated that a concentration as low as 0.2 µM for 24 h produced a similar effect on the inhibition of importin α nuclear to cytoplasmic distribution than a concentration of 2.5 µM for 1 h. This observation suggests that a sustained exposure to lower concentrations of IVM could indeed interfere with the host cell machinery that the virus requires for replication. Interestingly, results from our clinical trial using a high dose of IVM of 0.6 mg/kg/day for five consecutive days in hospitalized COVID-19 patients demonstrated a concentration-dependent effect on the viral load of respiratory secretions. The viral decay rate was significantly greater in patients with IVM plasma levels of 160 ng/mL or higher [7], a concentration close to the one we observed in vitro effects on importin α nuclear localization (0.2 µM that corresponds to a concentration of 175 ng/mL).

A recent work by Kern et al. described an in silico model of SARS-CoV-2 viral kinetics with acquired immune response to analyze the dynamic impact of different regimens using repurposed drugs for COVID-19 treatment [37]. They found greatest effects for IVM, while other drugs such as hydroxychloroquine and lopinavir/ritonavir had little to no appreciable effect. However, it is important to note that according to this simulation study, IVM at 0.6 mg/kg/day may have prominent antiviral effects, whereas lower doses of 0.3 mg/kg/day have marginal efficacy [36], which might explain the results of recently published randomized clinical trials that found no clinically significant effects compared to the untreated controls [38]. Since patient safety with multiple-day high-dose IVM regimens was shown [7], the design of further trials to confirm antiviral and clinical efficacy as well as to explore factors involved in oral bioavailability of the drug, seem to be warranted. Nasal spray administration is other attractive alternate route that would allow IVM accumulation in nasopharyngeal tissue [39].

We have also showed that ATV at an in vitro concentration of 10 µM for 24 h is able to reduce importin α nuclear localization. Moreover, ATV seemed to increase the inhibitory effect of IVM on importin α in certain experimental conditions. Cholesterol depletion produced by lipophilic statins causes the shutdown of host cell signaling events requiring membrane localization such as the Rho GTPases RhoA and CDC42 [9]. In this regard, several functions that are relevant to viral pathogenesis including actin organization and intracellular transport depend on Rho GTPase signaling [40]. This may explain the observed additive effect of ATV and IVM on importin α cellular distribution. Accordingly, during the in vitro simulated viral infection, combined treatment using ATV plus IVM as well as both monotherapies separately were capable of reverting Poly(I:C)-induced NF-κB increased expression in A549 cells. Despite ATV, alone or combined with IVM, was shown to increase RhoA mRNA expression in Poly(I:C)-transfected cells, variable results were obtained after analyzing the expression profiles of other Rho GTPases in cells exposed to different compounds. We know that each Rho GTPase is involved in specific events related to cell signaling, motility, and intracellular trafficking. In this regard, RhoA mainly participates in the regulation of endocytosis, Rac1 mediates exocytic transport by interaction with different effector molecules, and CDC42 is a key regulator of vesicle trafficking [9]. These contrasting results, involving up- or downregulation of different Rho GTPase gene expression levels, could be interpreted as specific cellular adaptation mechanisms in response to the effects of the tested drugs. Our work has several limitations that should be noted. Regarding the bioinformatics analysis, access to complete clinical records were unavailable, hence correlation analyses between gene expression and illness severity or comorbidities could not be performed. Additionally, raw RNA-Seq data were unavailable, thus we were not able to perform the pre-processing and alignment of sequences locally. We could, however, curate the pre-processed data by removing samples with >70% of total genes with 0 sequence reads, considering them as very low-quality samples that might introduce a bias.

Additional limitations may arise from the selection of Poly(I:C) to mimic viral infection as opposed to a cell-based infection system when evaluating compounds as potential treatments for SARS-CoV-2 infections. However, extensive reports have provided sound evidence for the use of this model, showcasing how it imitates in vivo responses of the human lung to viral infection, and how it is currently used to assess expression alterations of host cell receptors associated with SARS-CoV-2 [41,42,43,44].

We also acknowledge that we have only focused on analyzing in vitro drug effects on host cell processes. Future studies should be performed in A549 lung epithelial cells and cell-based infection systems using SARS-CoV-2 to elucidate the mechanism of action of IVM and ATV upon infection. These studies could help to establish a direct association between drug impact on importin α nuclear transport and the actual reduction of viral load in vivo. Furthermore, other in vivo mechanisms of action of the drugs should be taken into consideration. Along with an increasing number of clinical trials assessing the clinical benefit of IVM for patients with COVID-19 [45], alternate immunological mechanisms have been proposed [35]. IVM has shown immunomodulatory and anti-inflammatory properties in mouse models of different diseases [46,47] as well as in SARS-CoV-2-infected hamsters [48]. Along the same line, statins have prominent in vivo effects on endothelial cell biology and it has been discussed whether they would be able to act on vascular dysfunction associated with COVID-19 [49]. Considering its role in hypertension and coronary disease, ATV was assessed retrospectively in severe COVID-19 patients having comorbidities, with good results in terms of reducing the need for mechanical ventilation and in-hospital mortality [50].

Our findings contribute to place IVM and ATV as interesting drugs to target importin-mediated nuclear trafficking upon SARS-CoV-2 infection that could potentially be translated to other infections such as Dengue fever, Zika, and Influenza, thus broadening the spectrum of host-centric antiviral drug repurposing.

## Figures and Tables

**Table 1:** Table of primers, containing the gene name, sequences, and annealing temperature (T° An.).

Gene	Forward (5′–3′)	Reverse (5′–3′)	T° An.
*PPIA*	GGTATAAAAGGGGCGGGAGG	CTGCAAACAGCTCAAAGGAGAC	60°
*NFKB*	ACTCGCCACCCGGCTTCAGA	GGGCCATCTGCTGTTGGCAGT	60°
*KPNA7*	AGGACATGGAGCTGAGAAGTC	GACTGACCGCCATCCTCTG	57°
*KPNA5*	GCATTAAGGGCAGTTGGT	CAGTCCAGCAGGCTTCTTT	57°
*KPNA2*	GTGGACCCTTTGAACGCAGT	TTGAATCTGTGAAGACGGGCA	60°
*RHOA*	AAGGACCAGTTCCCAGAGGT	AGCCAACTCTACCTGCTTTCC	58°
*RAC1*	CCCCCTATCCTATCCGCAAAC	AACACATCGGCAATCGGCTT	58°
*CDC42*	AGGCTGTCAAGTATGTGGAGTG	TCTTCTTCGGTTCTGGAGGC	60°

## Data Availability

The datasets used and/or analyzed during the current study are available from the corresponding author on reasonable request.

## Supplementary Materials

The following are available online at www.mdpi.com/article/10.3390/v13102084/s1; Table S1: Patient demographics for GSE152075 dataset. Figure S1: Gene expression in COVID-19 and non-COVID-19 patients categorized by sex. Figure S2: Gene expression stratified by viral load. Figure S3: Gene expression of importins and Rho GTPases in A549 cells and ferrets. Figure S4: Importin and Rho GTPases gene expression analysis in A549 and HMVEC cells. Figure S5: Effect of high IVM concentrations on cell growth. Figure S6: Nuclear accumulation of importin α in response to oxidative stress. Figure S7: Effect of short-term exposure ATV on importin α nuclear accumulation. Figure S8: Impact of IVM at low concentration on nuclear accumulation of pp53. Figure S8: Impact of IVM at low concentration on nuclear accumulation of pp53.
