# Supplementary material for: Effect of Ivermectin and Atorvastatin on Nuclear Localization of Importin Alpha and Drug Target Expression Profiling in Host Cells from Nasopharyngeal Swabs of SARS-CoV-2- Positive Patients
Source: Viruses. 2021 Oct 15;13(10):2084. doi: 10.3390/v13102084 (PMC8537229; doi:10.3390/v13102084)
Supplement: Supplementary file 1 [file viruses-13-02084-s001.zip › Figure S1.pdf]

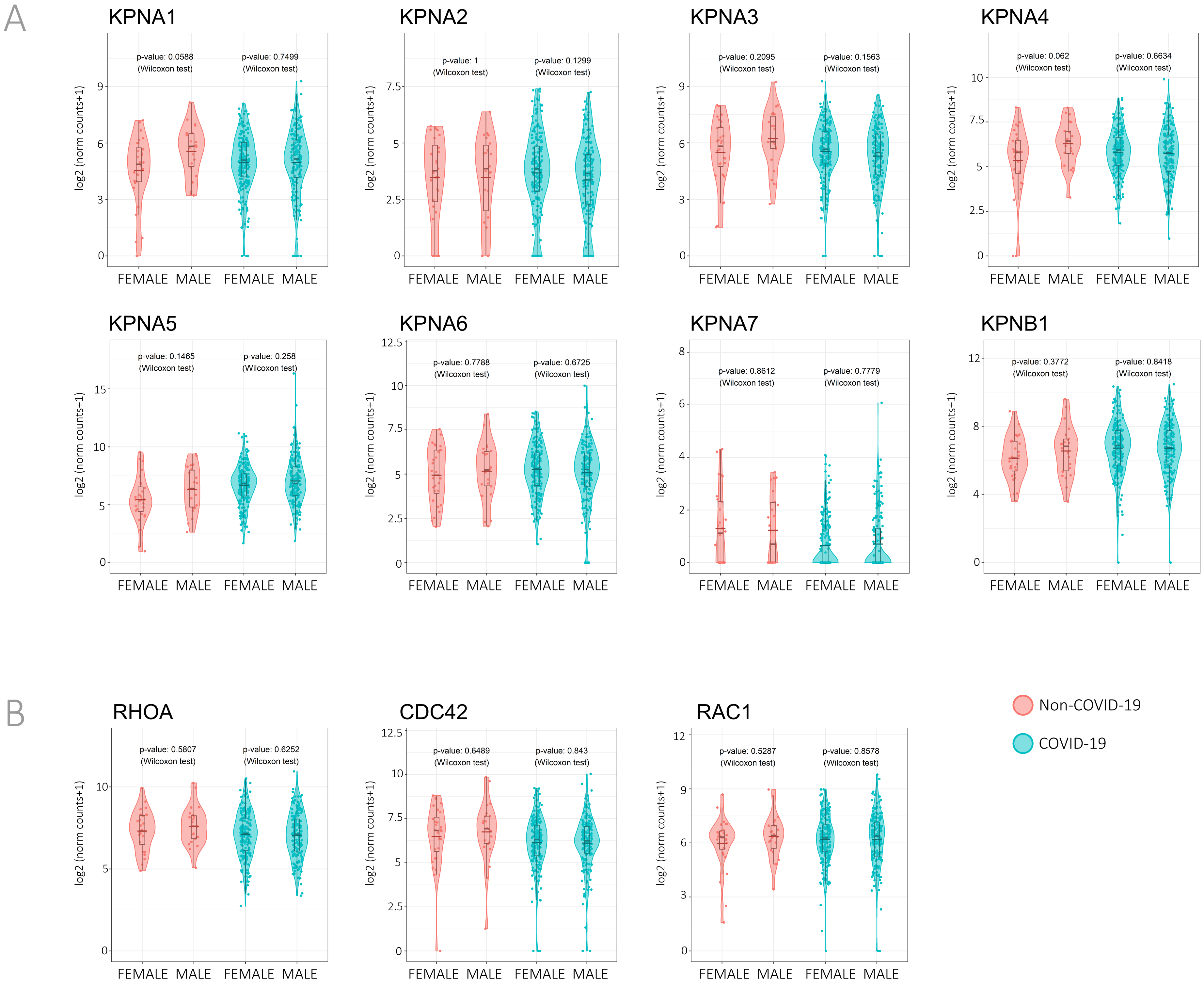

Figure S1: Gene expression in COVID-19 and non-COVID-19 patients categorized by sex.
A. Gene expression analysis for the importin family genes comparing COVID-19 and non-COVID19 patients from GSE152075 dataset categorized by sex. **B.** Gene expression analysis for the Rho-GTPase family genes comparing COVID-19 and non-COVID19 patients from GSE152075 dataset categorized by sex.
