# Supplementary material for: Effect of Ivermectin and Atorvastatin on Nuclear Localization of Importin Alpha and Drug Target Expression Profiling in Host Cells from Nasopharyngeal Swabs of SARS-CoV-2- Positive Patients
Source: Viruses. 2021 Oct 15;13(10):2084. doi: 10.3390/v13102084 (PMC8537229; doi:10.3390/v13102084)
Supplement: Supplementary file 1 [file viruses-13-02084-s001.zip › Figure S2.pdf]

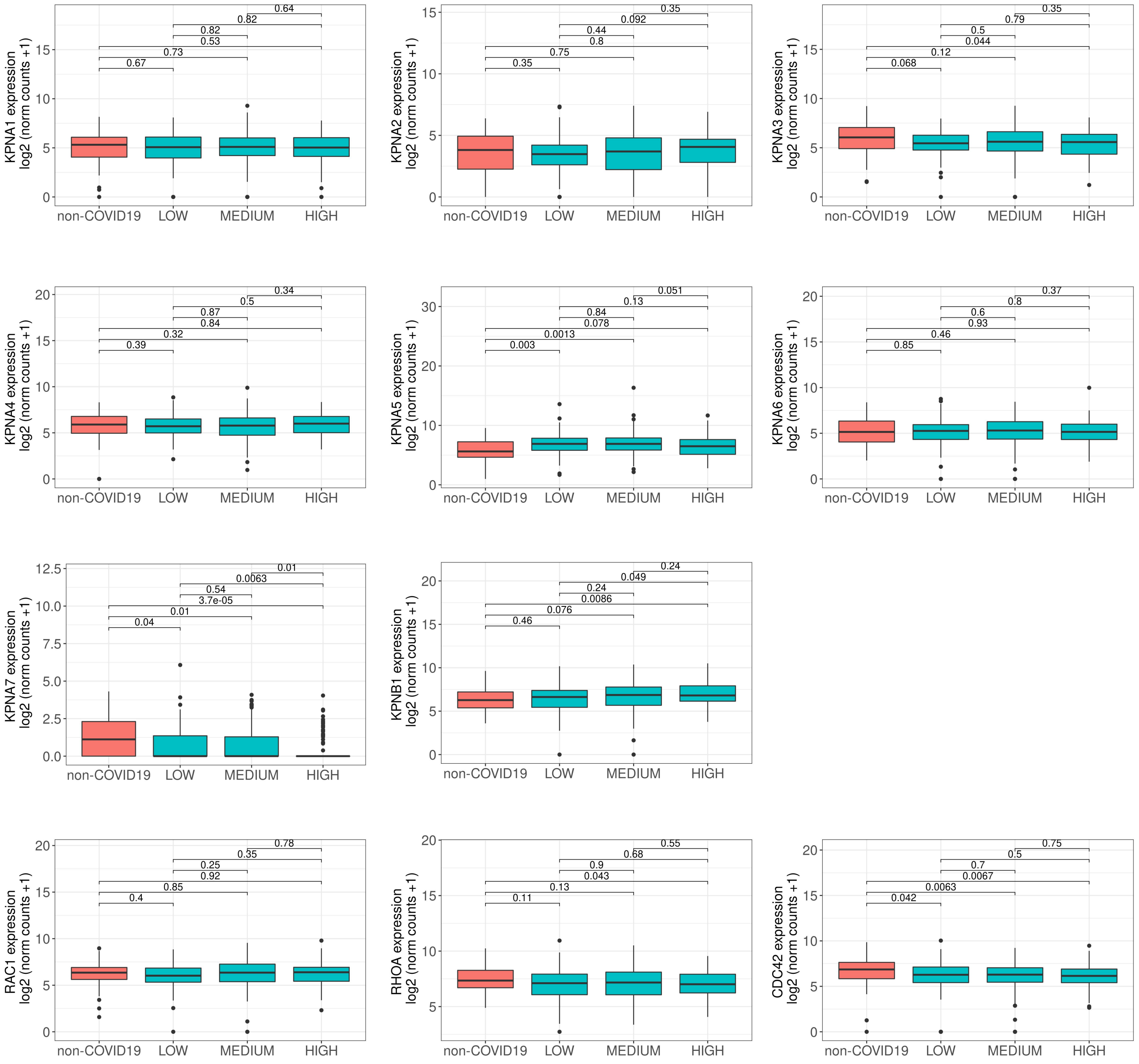


**Figure S2. Gene expression stratified by viral load.** Gene expression analysis of non-COVID-19 patients (red) and COVID-19 patients (blue) stratified by low (Ct > 24), medium (Ct = 24–19), and high (Ct < 19) viral load (GSE152075). Viral load was defined by the cycle threshold (Ct) of the N1 viral gene amplification during diagnostic PCR. (p values correspond to pairwise Wilcoxon rank-sum test)
