# Supplementary material for: Effect of Ivermectin and Atorvastatin on Nuclear Localization of Importin Alpha and Drug Target Expression Profiling in Host Cells from Nasopharyngeal Swabs of SARS-CoV-2- Positive Patients
Source: Viruses. 2021 Oct 15;13(10):2084. doi: 10.3390/v13102084 (PMC8537229; doi:10.3390/v13102084)
Supplement: Supplementary file 1 [file viruses-13-02084-s001.zip › Figure S3.pdf]

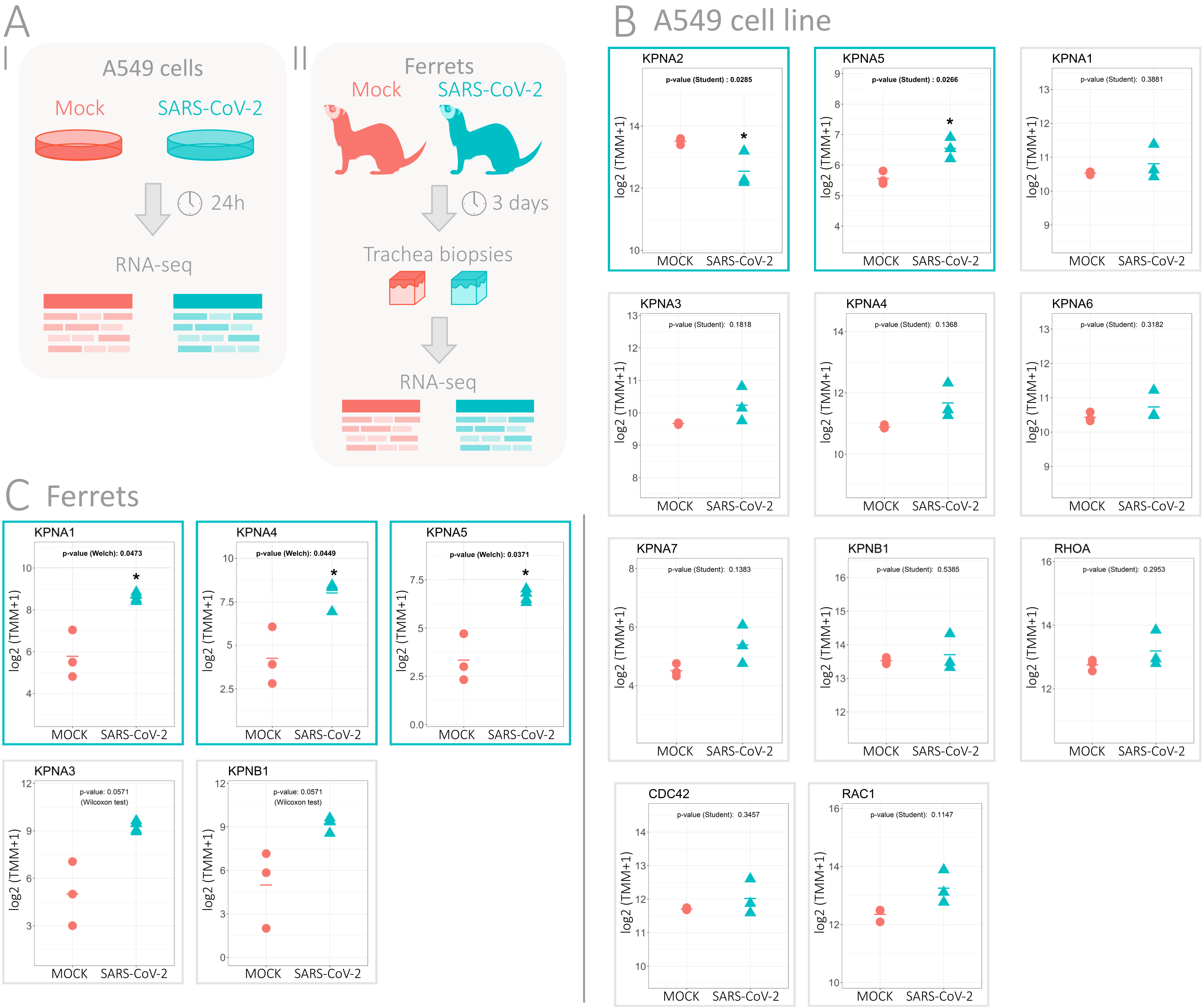


**Figure S3: Gene expression of importins and Rho-GTPases in A549 cells and ferrets.**
**A.** Experimental design of GSE147507 dataset, composed of I. A549 cells infected or not with SARS-CoV-2 for 24h with a multiplicity of infection (MOI) of 2 and II. trachea biopsies of 4-month-old ferrets harvested 3 days after infection with SARS-CoV-2 or mock treatment. **B.** Gene expression of importins and Rho-GTPases in A549 cell line. Statistical significance was calculated using Student's t test. **C.** Gene expression of importins in ferrets. Statistical significance was calculated using Welch´s t test.
