# Supplementary material for: Effect of Ivermectin and Atorvastatin on Nuclear Localization of Importin Alpha and Drug Target Expression Profiling in Host Cells from Nasopharyngeal Swabs of SARS-CoV-2- Positive Patients
Source: Viruses. 2021 Oct 15;13(10):2084. doi: 10.3390/v13102084 (PMC8537229; doi:10.3390/v13102084)
Supplement: Supplementary file 1 [file viruses-13-02084-s001.zip › Figure S4.pdf]

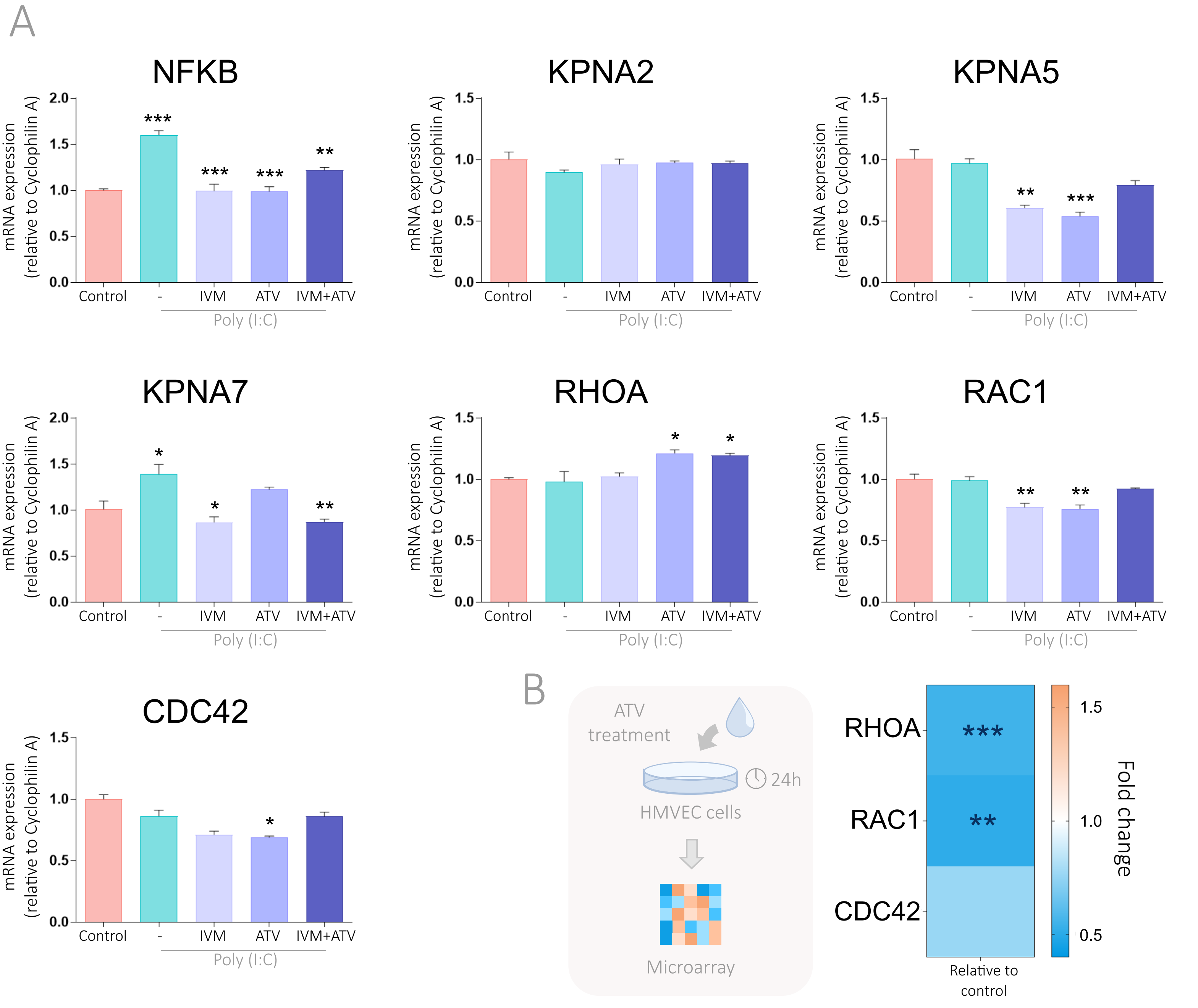

Figure S4. Importin and RhoGTPases gene expression analysis in A549 and HMVEC cells.
A. NFKB, KPNA2, KPNA5, KPNA7, RHOA, RAC1 and CDC42 expression levels assessed by real-time PCR (RT-qPCR) in A549 cells transfected with Poly (I:C) (10 µg/ml) and treated with PBS as control, or ivermectin (IVM; 2.5 µM), atorvastatin (ATV; 10 µM) or the combination of both drugs (ATV+IVM). Values were normalized using PPIA as a reference gene and relativized to control. Asterisks over PBS-treated bars indicate significant differences between control and Poly (I:C) conditions, wherase asteriscs over drug-treated bars indicate significant differences between ATV, IVM or ATV+IVM and Poly (I:C) conditions (*$p < 0.05$; **$p < 0.01$; ***$p < 0.001$). **B.** Heatmap depicting RHOA, RAC1 and CDC42 fold changes in mRNA levels assessed by microarray analysis of the GSE8686 dataset on human microvascular endothelial cells (HMVEC) treated or not with ATV (10 µM 24 h). Orange, white and blue represent a fold change > 1, fold change = 1 or fold change < 1, respectively. Statistical significance: *$p<0.05$; **$p<0.01$; ***$p<0.001$.
