# Supplementary material for: Effect of Ivermectin and Atorvastatin on Nuclear Localization of Importin Alpha and Drug Target Expression Profiling in Host Cells from Nasopharyngeal Swabs of SARS-CoV-2- Positive Patients
Source: Viruses. 2021 Oct 15;13(10):2084. doi: 10.3390/v13102084 (PMC8537229; doi:10.3390/v13102084)
Supplement: Supplementary file 1 [file viruses-13-02084-s001.zip › Figure S5.pdf]

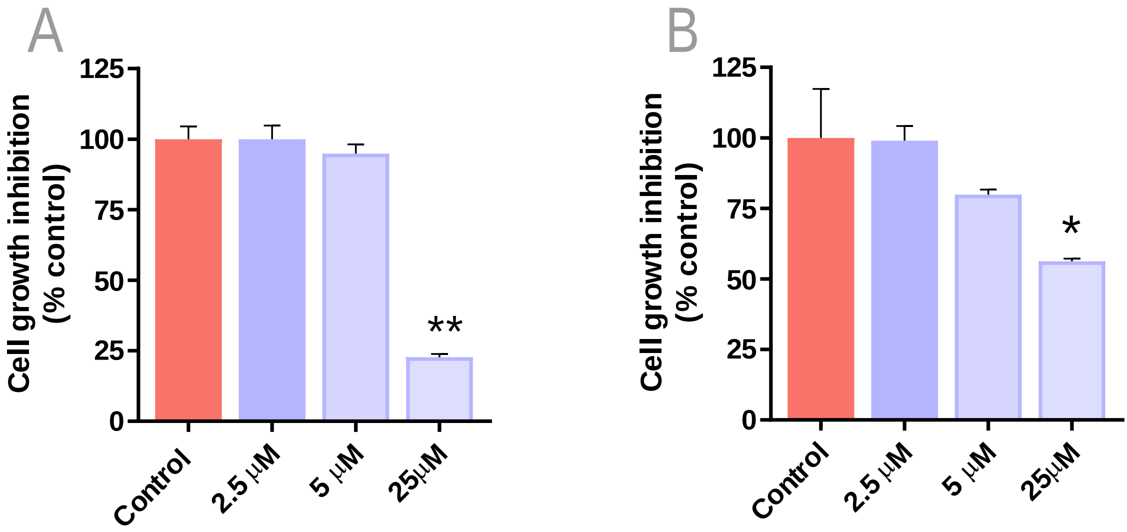

Figure S5. Effect of high IVM concentrations on cell growth.
(A) Hela and (B) Vero cells were treated with increased concentrations of IVM (2.5, 5 and 25 µM) and cell viability was evaluated after 48h-incubation. Data were normalized to control cells and are shown as mean ± SEM. Statistical significance: *$p < 0.05$; **$p < 0.01$ vs control group; Kruskal-Wallis followed by Dunn's multiple comparisons test.
