# Supplementary material for: Effect of Ivermectin and Atorvastatin on Nuclear Localization of Importin Alpha and Drug Target Expression Profiling in Host Cells from Nasopharyngeal Swabs of SARS-CoV-2- Positive Patients
Source: Viruses. 2021 Oct 15;13(10):2084. doi: 10.3390/v13102084 (PMC8537229; doi:10.3390/v13102084)
Supplement: Supplementary file 1 [file viruses-13-02084-s001.zip › Figure S6.pdf]

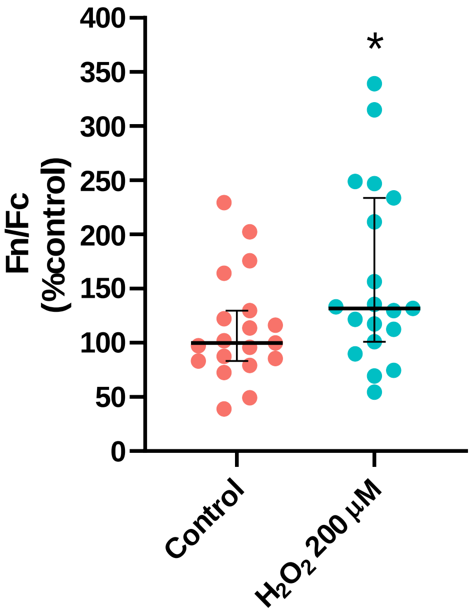

Figure S6. Nuclear accumulation of importin **α** in response to oxidative stress. Hela cells were incubated with 200 µM H_2O_2 and nuclear to cytoplasmic importin α distribution was evaluated by confocal microscopy. Each data point represents Fn/Fc from a single cell; data were normalized to control cells and are indicated as median with interquartile range. Statistical significance: $*p < 0.05$; Mann-Whitney test.
