# Supplementary material for: Effect of Ivermectin and Atorvastatin on Nuclear Localization of Importin Alpha and Drug Target Expression Profiling in Host Cells from Nasopharyngeal Swabs of SARS-CoV-2- Positive Patients
Source: Viruses. 2021 Oct 15;13(10):2084. doi: 10.3390/v13102084 (PMC8537229; doi:10.3390/v13102084)
Supplement: Supplementary file 1 [file viruses-13-02084-s001.zip › Figure S7.pdf]

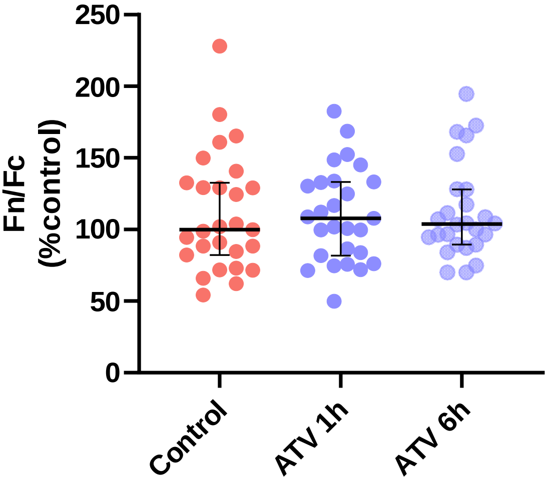

Figure S7. Effect of short term exposure ATV on importin **α** nuclear accumulation. Hela cells were treated with 10 μM ATV for 1 or 6 h and importin α cellular distribution was evaluated by confocal microscopy. Each data point represents Fn/Fc from a single cell; data were normalized to control cells and are indicated as median with interquartile range.
