# Supplementary material for: Effect of Ivermectin and Atorvastatin on Nuclear Localization of Importin Alpha and Drug Target Expression Profiling in Host Cells from Nasopharyngeal Swabs of SARS-CoV-2- Positive Patients
Source: Viruses. 2021 Oct 15;13(10):2084. doi: 10.3390/v13102084 (PMC8537229; doi:10.3390/v13102084)
Supplement: Supplementary file 1 [file viruses-13-02084-s001.zip › Figure S8.pdf]

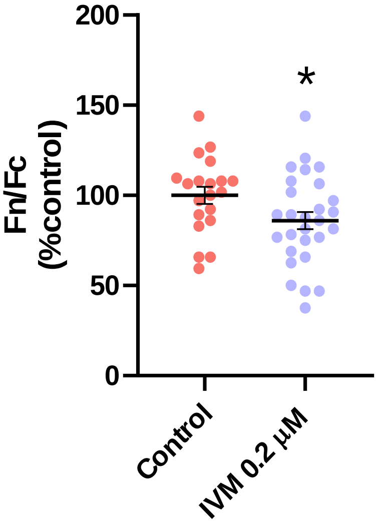

Figure S8. Impact of IVM at low concentration on nuclear accumulation of pp53. Hela cells were exposed to 0.2 µM IVM for 24 h and cellular distribution of pp53, one of the importin α cargo proteins, was evaluated. Each data point represents Fn/Fc from a single cell; data were normalized to control cells and are indicated as mean ± SEM. Statistical significance: *$p < 0.05$; Unpaired t test.
