# Supplementary material for: Effect of Ivermectin and Atorvastatin on Nuclear Localization of Importin Alpha and Drug Target Expression Profiling in Host Cells from Nasopharyngeal Swabs of SARS-CoV-2- Positive Patients
Source: Viruses. 2021 Oct 15;13(10):2084. doi: 10.3390/v13102084 (PMC8537229; doi:10.3390/v13102084)
Supplement: Supplementary file 1 [file viruses-13-02084-s001.zip › Table S1 - Patients demographics.pdf]

		Non-COVID-19	COVID-19	p-value
Number of patients		50	403	
Sex	Male	22 (44%)	166 (41.2%)	
	Female	28 (56%)	187 (46.4%)	0.763 [a]
	Unknown	-	50 (12.4%)	
Age	Range	12-91	2-91	
	Inter Quartile Range	29-63	41-71	
	Median	46.5	56	
	Mean	46.5	55.6	0.002 [b]
	< 30	13 (26%)	41 (10.2%)	
	30s	5 (10%)	51 (12.7%)	
	40s	10 (20%)	55 (13.6%)	
	50s	7 (14%)	80 (19.9%)	
	60s	8 (16%)	50 (12.4%)	
	≥ 70	7 (14%)	110 (27.3%)	0.012 [c]
	Unknown	-	16 (3.9%)	

Table S1. Patients demographics for GSE152075 dataset.

Table contains the number and % of COVID-19 and non-COVID-19 patients according to sex and age.

[a] Fisher's exact; [b] Student's t test; [c] Chi-square test.
